# Lamellar Keratoplasty: A Literature Review

**DOI:** 10.1155/2013/894319

**Published:** 2013-10-07

**Authors:** Ladan Espandar, Alan N. Carlson

**Affiliations:** Duke Eye Center, Durham, NC 27710, USA

## Abstract

The concept of lamellar keratoplasty (LK) is not new. However, newer forms of lamellar keratoplasty techniques have emerged in the last decade or so revolving around the concept of targeted replacement of diseased corneal layers. These include anterior lamellar keratoplasty (ALK) techniques that aim to selectively replace diseased corneal stroma and endothelial keratoplasty techniques aiming to replaced damaged endothelium in endothelial disorders. Recent improvements in surgical instruments and introduction of new techniques as well as inherent advantages such as preservation of globe integrity and decreased graft rejection have resulted in the reintroduction of LK as an acceptable alternative to conventional PK. In this review, indications, benefits, limitations, and outcomes of various anterior and posterior lamellar keratoplasty techniques are discussed.

## 1. Introduction

Until recently, penetrating keratoplasty (PK) or full-thickness corneal transplantation has been the most common surgical approach for the treatment of keratoconus, keratoectasia, and corneal scarring. By contrast, lamellar keratoplasty (LK) involves selective removal and replacement of diseased corneal layers. In this review, indications, benefits, limitations, and outcomes of various anterior and posterior lamellar keratoplasty techniques are discussed. Our literature review is derived from the Medline-PubMed databases from January 2000 February to 2013 using key words such as anterior lamellar keratoplasty, posterior lamellar keratoplasty, DSEK and DMEK. 

## 2. Anterior Lamellar Keratoplasty (ALK)

Lamellar keratoplasty (LK) targets partial or lamellar replacement of diseased corneal tissue. ALK preserves the posterior stroma. The advantages of ALK include reducing the risk of endothelial graft rejection, retaining structural integrity, and reducing potential intraoperative complications associated with open sky procedures [1]. However, manual dissection of the recipient bed and donor tissue potentially heals with interface scaring or haze that reduces the patient's quality of vision. More recently, improved instrumentation, surgical techniques, and automation have improved surgical efficiency and visual outcomes following ALK surgery. Studies now confirm that ALK visual outcomes are comparable to those of PK surgery [2] while achieving the advantages previously mentioned. In this section, we discuss various approaches addressing both superficial and deep ALK. 

## 3. Superficial Anterior Lamellar Keratoplasty (SALK) 

### 3.1. Indication

Anterior stromal scarring or opacification may result from stromal dystrophy, infection, inflammation, trauma, or previous surgery including refractive procedures. Removal of superficial lesions using manual dissection potentially leads to interface haze resulting from interface irregularities produced by surgical dissection. Phototherapeutic keratectomy (PTK) is capable of removing anterior scarring; however, it has several limitations. Scars frequently ablate differently than normal corneal it tissue, and a “masking” agent is needed to optimize smoothness. PTK may also heal with additional scarring, particularly when treating deeper lesions. Postoperatively, patients often need several months of low dose topical corticosteroids. Patients may also heal with a hyperopic shift, and even late scarring may develop following PTK treatment [3]. Herein, other methods of anterior stromal dissection: microkeratome, and femtosecond laser-assisted ALK are discussed in more detail. 

### 3.2. Surgical Techniques

*(A) Microkeratome-Assisted ALK*. A microkeratome is used to cut a superficial lamella (free cap) from the recipient cornea. A lamella of the same thickness is obtained from the donor cornea mounted on an artificial anterior chamber. The recipient bed is then measured with calipers, and the donor tissue is punched to the same size using a donor punch. The donor graft can be sutured into the recipient bed [4].

*(B) Sutureless Femtosecond Laser-Assisted Anterior Lamellar Keratoplasty (FALK)*. The depth of the recipient corneal pathology is measured using anterior segment OCT (AS-OCT). A femtosecond laser is used to create the lamellar cut in the recipient with donor corneas. Donor cut is adjusted according to the depth of the lesions with an additional 10–20% thickness adjusted to compensate for donor tissue swelling. The recipient's scarred corneal tissue is removed and replaced with the corneal donor lenticule. The incision is dried, and flap adhesion is checked. A bandage soft contact lens is placed over the cornea [5]. 

A modified method of FALK was reported by Bonfadini et al. using Ziemer femtosecond laser (Ziemer Ophthalmic Systems AG, Port, Switzerland) with 2 different dissection depths: midstromal (>250 *μ*m of posterior residual corneal bed thickness) and pre-Descemet (approximately 50 *μ*m of posterior residual corneal bed thickness) with the same principals described above [6].

### 3.3. Outcome

Patel et al. [4] performed microkeratome-assisted SALK in nine eyes of 8 consecutive patients with anterior stromal dystrophy recurrence (*n* = 3), post photorefractive keratectomy (PRK) haze (*n* = 2), and scarring after stromal melt (*n* = 4). They reported improved best corrected visual acuity (BCVA) in all 9 eyes at final followup with BCVA ≥ 20/40 in 7 of 9 eyes within the first month. The average follow-up period was 28 ± 3.9 months. Refractive astigmatism also improved by an average of 0.7 diopters. 

Shousha et al. [5] reported long-term outcome of FALK in thirteen consecutive patients. The BCVA significantly improved over preoperative values at the 12-, 18-, 24-, and 36-month visits. 54% of all patients had BCVA greater than 20/30 at the 12-month followup. Two patients lost a mean of 1.5 lines of BCVA because surface haze developed after PRK in one patient, and granular dystrophy recurred in the graft of the second patient. At the 12-month visit, mean spherical equivalent and refractive astigmatism were −0.4 diopters (D) and 2.2 D, respectively. Adjunctive procedures included PTK, PRK, cataract extraction, and epithelial ingrowth debridement. 

Bonfadini et al. [6] reported that uncorrected visual acuity (UCVA) and BCVA improved in all patients who underwent modified FALK compared with preoperative visual acuity, and all the eyes had clear grafts at the 1-year followup. The mean difference between preoperative and postoperative BCVAs was a gain of 8.0 lines. 

### 3.4. Complications

Complications such as residual corneal pathology, mild interface haze, anisometropia, recurrence of pathology, haze after adjunctive PRK, dry eye, epithelial ingrowth, and suspicious ectasia are reported in superficial FALK [5]. No graft failure or immunologic rejection episodes were noted in SALK [4–6].

## 4. Deep Anterior Lamellar Keratoplasty (DALK) 

### 4.1. Indication

DALK aims to remove and replace total or near-total corneal stroma while preserving host healthy endothelium. The advantages of DALK include reducing the risk of endothelial graft rejection [7], preservation of host endothelium with minimal surgical trauma [8], efficient visual rehabilitation relative to PK [9, 10], and also fewer intraoperative and postoperative complications including expulsive hemorrhage, anterior synechia, postoperative endophthalmitis, and glaucoma in comparison to PK. This procedure also requires less rigid criteria for donor corneal tissue selection that is often weighted toward donor endothelium in PK [11]. 

### 4.2. Surgical Techniques

*(A) Direct Open Dissection*. This method was first described by Anwar in 1972 [12]. Partial trephination is followed by lamellar dissection, originally accomplished using a rounded 69 Beaver blade and more recently with a Martinez dissecting spatula or a variety of dissecting blades. The dissection of deeper layers places Descemet's membrane (DM) at a greater risk for rupture.

*(B) Dissection with Hydrodelamination.* Sugita and Kondo described a technique of intrastromal fluid injection [13]. Partial trephination and lamellar keratectomy are followed by injecting saline into the stromal bed using a 27-gauge needle. Stromal swelling separates tissue making deeper dissection safer with respect to DM ruptures; however, perforation may still occur (39.2% in this study [13]). Some of the cases were successfully managed using air tamponade. 

*(C) Closed Dissection (Melles Technique).* Melles et al. described a closed dissection technique in 1999 that begins by exchanging aqueous with air [14]. This technique facilitates corneal dissection depth for the advancement of a specially designed spatula, thus creating a long, deep stromal pocket across the cornea. This pocket is further enlarged using sideways movements of the spatula and injection of viscoelastic [15]. A suction trephine blade is used to enter this viscopocket, and the stroma over the pocket is excised. A full-thickness donor button after removal of DM is sutured in place. Good visual results have been reported with this technique. DM perforation occurred in 14% of the cases reported [14]. 

*(D) Dissection with Anwar's Big Bubble Technique.* The big bubble technique described by Anwar and Teichmann in 2002 continues to gain popularity [16]. The cornea is trephined and dissected at a depth of approximately 60–80%. Air is injected paracentrally through a 27- or 30-gauge needle or specially designed cannula producing “big bubble” separation of DM from stroma. Entry into this space followed by removal of stromal tissue involves a process that meticulously protects and preserves DM. Same size or 0.25 mm oversized donor is sutured in place after donor DM is removed. 

*(E) Big Bubble Technique Combined with Femtosecond Laser Trephination.* The use of a femtosecond laser for the dissection of anterior lamella in anterior keratoplasty was first described by Suwan-Apichon et al. [17] in 2006 and later by Price Jr. et al. [18] and Farid and Steinert in 2009 [19]. The technique offers “zigzag” or “mushroom” configured wound construction in both patient and donor directed at reducing postoperative astigmatism, improving wound strength, and allowing earlier suture removal [18, 19]. 

*(F) Other Modifications*. Modifications to big bubble technique have been applied in unusual cases such as a cornea with 16 radial keratotomy incisions [20], descematocele [21], and healed hydrops [22]. 

### 4.3. Outcome

Several studies have shown that refractive spherical equivalent, BCVA, contrast sensitivity function (CSF), and higher order aberrations (HOAs) in DALK are comparable to PK especially in keratoconus patients. A clinical trial by Javadi et al. demonstrated that the mean postoperative spherical equivalent was −3.23 ± 3.4 diopters in the DALK group versus −2.22 ± 4.6 diopters in the PK group (*P* = 0.28). Mean postoperative BCVAS were 0.18 ± 0.08 and 0.15 ± 0.10 logarithm of the minimum angle of resolution (logMAR), respectively (*P* = 0.12). CSF and total aberrations and HOAs were comparable in both groups [23]. 

In another comparative study, Cohen et al. found comparable visual outcomes between PK and DALK; however, achieving 20/20 acuity was higher in the PK group [24]. 

Mashor et al. compared the visual and refractive outcomes between DALK and intralase enabled keratoplasty (IEK) for keratoconus. The mean logMAR BCVA of patients in the DALK group was 0.28 (20/38) and 0.37 (20/46) in the IEK group (*P* < 0.211). The final spherical equivalents were −5.62 and −0.53 in the DALK and IEK groups, respectively (*P* < 0.973). There was a statistically significant difference in the astigmatism between the 2 groups with mean manifest cylinder of 3.13 in the DALK group and 5.78 in the IEK group (*P* < 0.011). Total HOA (DALK 6.11 versus IEK 9.32, *P* < 0.036) and total spherical aberrations (DALK 0.44 versus IEK 0.71, *P* < 0.041) were both significantly higher in the IEK group [25]. The difference in vision in cases that retained a small layer stroma did not achieve statistical significance. Sarnicola et al. compared the outcome of Descemetic and pre-Descemetic DALK and showed that there was no difference in visual acuity between the pdDALK and dDALK groups at an average followup of 30.4 months, although the eyes in the dDALK group seemed to have faster visual recovery. BCVA was at least 20/30 in 80–85% of eyes at the patient's last visit in both groups [26]. 

In a similar study, Abdelkader and Kaufman showed that 90.9% of the Descemetic group achieved final BCVA of 20/30 or better. This compares with 75% of the pre-Descemetic group achieving this vision; however, this did not achieve statistical significance based on their numbers. They did however find on confocal examination that the reflectivity of activated keratocytes at the interface was less in the Descemetic than that in the pre-Descemetic group that retained host stroma. Ten to 12 weeks after pdDALK and 4 to 6 weeks after dDALK, keratocyte morphology and reflectivity had returned to normal [27]. 

### 4.4. Complications

*(A) Stromal Rejection.* Olson et al. reported 22 patients undergoing DALK who experienced stromal rejection manifested as acute stromal edema and/or stromal neovascularization. Five of these patients experienced their stromal rejection within 12 months. All episodes resolved completely with treatment [28].

Mosca et al. reported 3 cases of stromal rejection after femtosecond laser-assisted DALK. This presented as both superficial and deep neovascularization along with stromal edema and infiltration, all documented using confocal microscopy revealing cellular inflammatory infiltrates within the stroma. The inflammatory process and presumed graft rejection was rapidly reversed with topical steroid therapy, and a clear cornea was achieved in all cases [29].

*(B) Other Complications.* Fixed dilated pupil seen with the Urrets-Zavalia syndrome is an uncommon but serious complication of corneal transplantation. In this syndrome, the iris is fixed and dilated and may adhere to anterior lens capsule. Iris atrophy develops over time and is frequently associated with anterior subcapsular lens opacity resulting from metabolic disruption. DALK can decrease the risk of this complication; however, Maurino et al. reported three cases of fixed dilated pupil and iris ischemia (Urrets-Zavalia syndrome). This was presumably a response to elevated pressure causing ischemia from pupillary blockage from air in the anterior chamber [30]. Niknam and Rajabi reported 4 cases that developed fixed dilated pupil after performing DALK for keratoconus and granular corneal dystrophy [31]. 

## 5. Posterior Lamellar Keratoplasty or Endothelial Keratoplasty (EK) 

Attempting to replace endothelial pathology, the first posterior lamellar keratoplasty (PLK) procedure was described by Barraquer in 1950. Melles et al. [33] offered sutureless PLK in 1998, using an air bubble for graft fixation. In 2001, Terry and Ousley [34] introduced endothelial keratoplasty (EK) and deep lamellar endothelial keratoplasty (DLEK). Further improvements in EK were described in 2005 by Price Jr. and Price [35] who performed Descemet stripping endothelial keratoplasty (DSEK). A year later, Gorovoy [36] added automation using a microkeratome for Descemet stripping automated endothelial keratoplasty (DSAEK). Subsequently, Descemet membrane endothelial keratoplasty (DMEK) was described by Melles et al. [37] allowing transplantation of an isolated endothelium-Descemet membrane layer (EDM) without adherent corneal stroma. Presently, DMEK offers the best approximation and opportunity to return the patient to normal anatomic configuration.

### 5.1. DSAEK/DSEK 

#### 5.1.1. Indication

DSAEK is beneficial in treating patients with Fuchs' endothelial dystrophy and other forms of corneal decompensation resulting from endothelial cell loss [38]. This would also include genetic diseases such as iridocorneal endothelial syndrome [39, 40] and congenital hereditary endothelial dystrophy [41]. 

#### 5.1.2. Surgical Techniques

The classic technique for DSAEK consists of a 4-5 mm limbal or corneoscleral incision used for insertion of the donor tissue with forceps [42]. Several recent tissue inserter devices make the insertion possible through a smaller incision [43, 44]. Descemet stripping is performed for a diameter of 8.0 mm with a reverse-bent Sinskey hook corresponding to the 8.0-mm epithelial trephine marker. The recipient's endothelium and Descemet membrane are carefully removed [42]. Donor tissue may be prepared during surgery or “precut” by an Eye Bank facility. Pre-cut tissue uses either a microkeratome or a femtosecond laser. The microkeratome cutting depth is adjustable to a depth of 350 *μ*m, which generally prepares a donor tissue of 150–200 *μ*m in thickness. Clinical outcomes support the use of eye-bank-prepared tissue. Price et al. [45] found no difference in endothelial cell (EC) loss at 1 year in donor tissue prepared by an eye bank versus a surgeon. This study also reported similar visual outcomes and donor detachment rates. Other studies have also shown that the clinical outcomes and rate of EC loss in precut tissues are comparable to surgeon-prepared tissue [46]. Then donor tissue is trephined to the appropriate size, most commonly 8.0–8.5 mm. Donor tissue insertion into the anterior chamber may use several methods including forceps, Busin glide, cystotome, suture pull-through, and a variety of new inserters designed to provide a more delicate method of insertion. Air was carefully injected into the anterior chamber to unfold the graft. Ten minutes after the air injection, most of the air was replaced with balanced salt solution. 

Sikder et al. in an experimental laboratory investigation utilized a double pass microkeratome technique—a deeper cut followed by a thinner cut producing a donor thickness consistently less than 120 *μ*m [47]. 

Phillips et al. in an experimental study showed that Ziemer LDV, low pulse energy, and high-frequency femtosecond laser can safely prepare ultrathin cuts (70 *μ*m lenticule) with minimal endothelial cell damage; however, the resulting stromal surface quality may not be optimal with this technique [48]. 

#### 5.1.3. Outcome

The mean acuity after DSAEK is about 20/40, if eyes with visual comorbidities such as retinal disease or glaucoma are excluded [38]. The explanation for 20/40 acuity is presumably on the basis of interface light scatter at the tissue interface. However, Baratz et al. recently quantified corneal light scatter and its relationship to vision after DSEK and found that visual function after DSEK is also affected by residual haze in the anterior host cornea, and this may be more impactful than the surgical interface [49]. 

 Donor thickness contribution to visual outcome is somewhat controversial. Thicker tissue has not been found to significantly degrade BCVA outcome [50]. van der Meulen et al. evaluated the correlation of stray light and visual function in DSEK patients and did not find any correlation between corneal thickness with BCVA or straylight analysis. Corneal thickness and haze did not offer an adequate explanation for the decreased visual quality either [50]. Woodward et al. studied the relationship of visual acuity and lamellar thickness in sixty-four eyes of 52 patients with a mean followup of 27 ± 16 months who underwent DSEK and concluded that there was a poor correlation between visual acuity with preoperative or postoperative DSAEK graft thickness [51]. 

The magnitude of hyperopic shift appears to be about 0.8 to 1.5 diopters resulting from the “minus meniscus lens” contribution from the donor tissue [38]. 

Yamaguchi et al. evaluated the irregularity of the anterior and posterior cornea after DSAEK and PK using a rotating Scheimpflug camera and found that the regular astigmatism and tilt components of the anterior surface were significantly lower after DSAEK than after PK, whereas a statistically significant difference did not exist when comparing the posterior surfaces. Higher order aberrations (third- to eighth order components) of the anterior surface were significantly greater after PK in comparison to DSAEK, whereas there were no significant differences between the two groups when analyzing the posterior surface for higher order aberrations [52].

#### 5.1.4. Complications

The two most common early complications following DSAEK surgery are graft dislocation and primary graft failure. Both correlate with surgical technique and surgeon experience.

*(A) Primary Graft Failure.* Reported rates of primary graft failure (PGF) after DSAEK vary between 0% and 29%. It seems to be related to surgical technique, surgeon experience, and surgical complexity (e.g., presence of anterior chamber intraocular lens, large iridectomies and filtering tubes) [38]. 

*(B) Graft Dislocation.* The most common early complication following DSAEK surgery is dislocation of the graft, requiring another air bubble to reattach the tissue. The rate of donor dislocation has been reported between 1% and 82% [38]. 

The precise mechanism of donor adhesion is poorly understood. The risk of dislocation does not appear to be correlated with any donor characteristic. There does not appear to be an association of low endothelial cell density. Initially, there was a concern about tissue in storage media for a prolonged period; however, that has not been consistent even when using tissue stored in cold Optisol for up to 8 days. Eye rubbing may cause donor dislocation even 7 days after surgery. A major contributor to graft dislocation is hypotony (IOP < 5 mmHg) [38]. Dislocation is reduced by avoiding hypotony, reducing interface fluid with “venting incisions,” and some recommend a peripheral scratching of the posterior bed to facilitate adherence [38].

*(C) Rejection.* The rejection and failure rate following DSAEK surgery may be lower than that of PK resulting from better preservation of the ocular surface, absence of corneal sutures, and overall less inflammation postoperatively. It is reported between 7.5 and 9% [38].

*(D) Other Complications of DSAEK Surgery. *Other potential complications following DSAEK surgery include iatrogenic pupillary block glaucoma from the residual air bubble, interface epithelial ingrowth, corneal infection, and endophthalmitis [38]. 

Price's group reported five cases of visually significant haze in the graft-host interface presenting as a fine reticular pattern. Clinical examination and imaging supports the haze resulted from retained viscoelastic in the interface. Patients were either observed or underwent surgery to irrigate the viscoelastic from the interface [53]. 

Uncommon complications of DSAEK include dislocations of the donor graft into the posterior segment. Afshari et al. reported eight cases of posterior segment migration. Each eye had a history of vitrectomy. Five eyes had sutured posterior chamber intraocular lenses, 1 eye had a sulcus intraocular lens, and 2 eyes were aphakic. Each eye required repeat grafting, and in 6 of 8 eyes, pars plana vitrectomy was used to remove the dislocated graft. Final visual acuities ranged from 20/30 to no light perception [54]. 

### 5.2. DMEK

More recent modifications of  EK have attempted to transplant only donor DM and have been named DMEK by Melles et al. [37] and Descemet membrane automated endothelial keratoplasty (DMAEK) by Price et al. [55]. 

#### 5.2.1. Surgical Techniques

Immediately before transplantation, the DM is stripped from the donor corneal stroma in the following manner: after mounting the corneoscleral buttons on a suction trephine the endothelium is gently marked with an 8.0-mm trephine and stained with 0.06% trypan blue. The submerged DM peripheral to the mark is incised with a sharp blade. The central edge is first lifted with a round blade and then grasped with 2 forceps. An incomplete detachment of the DM is created through a simultaneous centripetal movement of the 2 forceps. This is followed by trephination with an 8.0 mm trephine. Transfer of the graft into the patient's eye and the unfolding were achieved by a standardized technique: the DM is placed into a customized glass injector (Melles) or a variety of modified intraocular lens injectors. Recipient preparation is done through a small corneal incision (approximately 2.5 mm), and the patient's DM is removed under air using an inverted hook. The graft is injected into the AC. The DM is then positioned centrally using short bursts of balanced salt solution and unfolded by injecting a series of small air bubbles. Once the donor is completely and properly unfolded, air is then injected underneath the graft until the AC is completely filled. The air is left in place for 30 minutes. On completion of the procedure, air is aspirated, decreasing to approximately 50% of the AC volume [32]. 

#### 5.2.2. Outcome

Kruse's group compared the visual outcome and endothelial cell survival in patients undergoing DMEK with those undergoing DSAEK and showed that BCVA increased from 0.70 ± 0.48logMAR and 0.75 ± 0.32logMAR before surgery to 0.17 ± 0.12logMAR and 0.36 ± 0.15logMAR 6 months after DMEK and DSAEK, respectively. Endothelial cell density decreased from 2575 ± 260 cells/mm^2^ and 2502 ± 220 cells/mm^2^ before surgery to 1520 ± 299 cells/mm^2^ and 1532 ± 495 cells/mm^2^ 6 months after DMEK and DSAEK, respectively. Central corneal thickness decreased from 652 ± 92 *μ*m before surgery to 517 ± 45 *μ*m 6 months after DMEK and from 698 ± 137 *μ*m before surgery to 618 ± 66 *μ*m 6 months after DSAEK and concluded that DMEK provided faster and more complete visual rehabilitation when compared with DSAEK [32]. 

Price's group evaluated outcomes of DSAEK in one eye and DMEK in the other eye of the same patient. At 12 months postoperatively, the mean BCVA in the DMEK group was 0.07logMAR (20/24) and 0.20logMAR (20/32) in the DSAEK group. The majority of the patients (85%) perceived better visual quality in the DMEK eye. Furthermore, 62% preferred or would recommend DMEK to a friend or relative, whereas 15% preferred DSAEK, and 23% reported no preference between the surgical procedures. The 1-year endothelial cell loss and the perceived discomfort level during the postoperative period were comparable for the 2 procedures. Faster recovery and better final visual acuity were the main benefits of the DMEK technique [56]. 

Melles' group determined the clinical outcomes of isolated DMEK in phakic eyes and showed that 85% reached equal to or better than 20/25 at 6 months. Mean endothelial cell density at 6 months was 1660 ± 470 cells/mm^2^. The final refractive result was a minor hyperopic shift (+0.74 diopter), and graft detachment rate was 4%. Temporary mechanical angle closure glaucoma due to air bubble dislocation behind the iris was the main complication (11.5%). Two eyes (4%) required phacoemulsification after DMEK [57]. 

Rudolph et al. compared corneal higher-order aberrations (HOAs) in eyes after DMEK, DSAEK, and PK and in a control group that had not undergone surgery. There were no statistically significant differences of HOAs for the anterior 4.0 mm zones between the DMEK group and the controls or between the DMEK and DSAEK groups. The DMEK procedure compared favorably with PK showing statistically significant differences in all terms for the 4.0-mm zones. All Zernike terms for mean posterior aberrations of the central 4.0-mm zones showed statistically significant higher aberrations for DMEK compared with controls. The DMEK procedure compared with DSAEK showed statistically significant lower mean values for all combined Zernike terms, except for coma. Compared with PK, DMEK showed statistically significant lower mean values for all combined Zernike terms for the central 4.0-mm zones of the posterior corneal surface, except for spherical aberration (SA). BCVA after DMEK was statistically significantly better than after DSAEK (*P* < 0.001) and PK (*P* < 0.005). There was no statistically significant difference when BCVA was compared with controls (*P* < 0.998) [58]. 

Melles' group evaluated twelve eyes of 12 patients, who underwent secondary DMEK to manage poor visual outcome after initial DSAEK. They found that four causes of reduced optical quality of the transplanted host cornea could be identified in DSAEK: five eyes (42%) showed large host-Descemet remnants within the visual axis during surgery; six eyes (50%) irregular graft thickness; six eyes subtle stromal waves'; and nine eyes (75%) high reflectivity at the donor-to-host interface. After DMEK graft replacement, all corneas cleared and achieved a BCVA of 20/25, except for one with a partial Descemet graft detachment. Pachymetry values decreased from 670 ± 112 *μ*m before to 517 ± 57 *μ*m after secondary DMEK. Higher order aberrations (Coma and Trefoil) at the posterior surface tended to be lower (*P* = 0.07) in DMEK grafts than in DSAEK grafts [59]. 

#### 5.2.3. Complications

*(A) Graft Rejection.* Melles' group reported the incidence of early allograft rejection after DMEK in the first series of 120 eyes of 105 patients operated on for Fuchs endothelial dystrophy or pseudophakic bullous keratopathy, with an average followup 2 years of. Only 1 of the eyes showed signs of a cellular immune response to the Descemet graft. Intensive topical corticoid therapy resulted in a complete visual recovery to 20/25 within weeks [60]. 

Price's group evaluated the relative risk of immunologic rejection in patients who underwent DMEK, DSEK, and PK. They compared one hundred forty-one eyes treated with DMEK retrospectively with cohorts of DSEK (*n* = 598) and PK (*n* = 30) patients. Only 1 patient (0.7%) had a documented rejection episode in the DMEK group compared with 54 (9%) in the DSEK and 5 (17%) in the PK group. The Kaplan-Meier cumulative probabilities of a rejection episode at 1 and 2 years were 1% and 1%, respectively, for DMEK; 8% and 12%, respectively, for DSEK; and 14% and 18%, respectively, for PK. Therefore, patients undergoing DMEK had a significantly reduced risk of experiencing a rejection episode within 2 years after surgery compared with DSEK and PK performed for similar indications using the same corticosteroid regimen [61]. 

*(B) Glaucoma.* The incidence of glaucoma was evaluated in the first 275 consecutive eyes that underwent DMEK for Fuchs endothelial dystrophy (260 eyes) or bullous keratopathy (15 eyes) by Melles. Glaucoma was defined as a postoperative intraocular pressure (IOP) elevation of >24 mmHg, or >10 mmHg from the preoperative baseline. 18 eyes (6.5%) showed postoperative glaucoma after DMEK. Seven eyes (2.5%) had an exacerbation of a preexisting glaucoma. Eleven eyes (4%) presented with a de novo IOP elevation, associated with air-bubble-induced mechanical angle closure (2%), steroid response (0.7%), or peripheral anterior synechia (0.4%), or without a detectable cause (0.7%). Two eyes (0.7%) required glaucoma surgery after DMEK. At 6 months, all eyes had a BCVA of >20/40, and 81% reached >20/25. Glaucoma after DMEK may be a relatively frequent complication that could be avoided by reducing the residual postoperative air bubble to 30% in phakic eyes, applying a population-specific steroid regimen, and avoiding decentration of the Descemet graft. Eyes with a history of glaucoma may need close IOP monitoring in the first postoperative months, especially in eyes with an angle-supported phakic intraocular lens [62].

## 6. Summary

Selectively approaching corneal pathology with lamellar surgery is a trend that will continue eventually making full-thickness penetrating keratoplasty an uncommon procedure.

## References

[1] Yeung SN, Lichtinger A, Kim P, Amiran MD, Rootman DS (2012). Retrospective contralateral study comparing deep anterior lamellar keratoplasty with penetrating keratoplasty: a patient's perspective. *Canadian Journal of Ophthalmology*.

[2] Tan DT, Mehta JS (2007). Future directions in lamellar corneal transplantation. *Cornea*.

[3] Rapuano CJ (2001). Excimer laser phototherapeutic keratectomy. *Current Opinion in Ophthalmology*.

[4] Patel AK, Scorcia V, Kadyan A, Lapenna L, Ponzin D, Busin M (2012). Microkeratome-assisted superficial anterior lamellar keratoplasty for anterior stromal corneal opacities after penetrating keratoplasty. *Cornea*.

[5] Shousha MA, Yoo SH, Kymionis GD (2011). Long-term results of femtosecond laser-assisted sutureless anterior lamellar keratoplasty. *Ophthalmology*.

[6] Bonfadini G, Moreira H, Jun AS (2013). Modified femtosecond laser-assisted autureless anterior lamellar keratoplasty. *Cornea*.

[7] Terry MA (2000). The evolution of lamellar grafting techniques over twenty-five years. *Cornea*.

[8] Morris E, Kirwan JF, Sujatha S, Rostron CK (1998). Corneal endothelial specular microscopy following deep lamellar keratoplasty with lyophilised tissue. *Eye*.

[9] Akdemir MO, Kandemir B, Sayman IB, Selvi C, Kamil Dogan O (2012). Comparison of contrast sensitivity and visual acuity between deep anterior lamellar keratoplasty and penetrating keratoplasty in patients with keratoconus. *International Journal of Ophthalmology*.

[10] Watson SL, Ramsay A, Dart JK, Bunce C, Craig E (2004). Comparison of deep lamellar keratoplasty and penetrating keratoplasty in patients with keratoconus. *Ophthalmology*.

[11] Luengo-Gimeno F, Tan DT, Mehta JS (2011). Evolution of deep anterior lamellar keratoplasty (DALK). *The Ocular Surface*.

[12] Anwar M (1972). Dissection technique in lamellar keratoplasty. *The British Journal of Ophthalmology*.

[13] Sugita J, Kondo J (1997). Deep lamellar keratoplasty with complete removal of pathological stroma for vision improvement. *The British Journal of Ophthalmology*.

[14] Melles GR, Rietveld FJR, Beekhuis WH, Binder PS (1999). A technique to visualize corneal incision and lamellar dissection depth during surgery. *Cornea*.

[15] Melles GR, Remeijer L, Geerards AJ, Beekhuis WH (2000). A quick surgical technique for deep, anterior lamellar keratoplasty using visco-dissection. *Cornea*.

[16] Anwar M, Teichmann KD (2002). Deep lamellar keratoplasty: surgical techniques for anterior lamellar keratoplasty with and without baring of Descemet’s membrane. *Cornea*.

[17] Suwan-Apichon O, Reyes JM, Griffin NB, Barker J, Gore P, Chuck RS (2006). Microkeratome versus femtosecond laser predissection of corneal grafts for anterior and posterior lamellar keratoplasty. *Cornea*.

[18] Price FW, Price MO, Grandin JC, Kwon R (2009). Deep anterior lamellar keratoplasty with femtosecond-laser zigzag incisions. *Journal of Cataract and Refractive Surgery*.

[19] Farid M, Steinert RF (2009). Deep anterior lamellar keratoplasty performed with the femtosecond laser zigzag incision for the treatment of stromal corneal pathology and ectatic disease. *Journal of Cataract and Refractive Surgery*.

[20] Chamberlain W, Cabezas M (2011). Femtosecond-assisted deep anterior lamellar keratoplasty using big-bubble technique in a cornea with 16 radial keratotomy incisions. *Cornea*.

[21] Sharma N, Kumar C, Mannan R, Titiyal JS, Vajpayee RB (2010). Surgical technique of deep anterior lamellar keratoplasty in Descemetoceles. *Cornea*.

[22] Ramamurthi S, Ramaesh K (2011). Surgical management of healed hydrops: a novel modification of deep anterior lamellar keratoplasty. *Cornea*.

[23] Javadi MA, Feizi S, Yazdani S, Mirbabaee F (2010). Deep anterior lamellar keratoplasty versus penetrating keratoplasty for keratoconus: a clinical trial. *Cornea*.

[24] Cohen AW, Goins KM, Sutphin JE, Wandling GR, Wagoner MD (2010). Penetrating keratoplasty versus deep anterior lamellar keratoplasty for the treatment of keratoconus. *International Ophthalmology*.

[25] Mashor RS, Rootman DB, Bahar I, Singal N, Slomovic AR, Rootman DS (2011). Outcomes of deep anterior lamellar keratoplasty versus intralase enabled penetrating keratoplasty in keratoconus. *Canadian Journal of Ophthalmology*.

[26] Sarnicola V, Toro P, Gentile D, Hannush SB (2010). Descemetic dalk and preDescemetic dalk: outcomes in 236 cases of keratoconus. *Cornea*.

[27] Abdelkader A, Kaufman HE (2011). Descemetic versus pre-Descemetic lamellar keratoplasty: clinical and confocal study. *Cornea*.

[28] Olson EA, Tu EY, Basti S (2012). Stromal rejection following deep anterior lamellar keratoplasty: implications for postoperative care. *Cornea*.

[29] Mosca L, Fasciani R, Mosca L (2011). Graft rejection after femtosecond laser-assisted deep anterior lamellar keratoplasty: report of 3 cases. *Cornea*.

[30] Maurino V, Allan BD, Stevens JD, Tuft SJ (2002). Fixed dilated pupil (urrets-zavalia syndrome) after air/gas injection after deep lamellar keratoplasty for keratoconus. *American Journal of Ophthalmology*.

[31] Niknam S, Rajabi MT (2009). Fixed dilated pupil (urrets-zavalia syndrome) after deep anterior lamellar keratoplasty. *Cornea*.

[32] Tourtas T, Laaser K, Bachmann BO, Cursiefen C, Kruse FE (2012). Descemet membrane endothelial keratoplasty versus Descemet stripping automated endothelial keratoplasty. *American Journal of Ophthalmology*.

[33] Melles GR, Eggink FA, Lander F (1998). A surgical technique for posterior lameliar keratoplasty. *Cornea*.

[34] Terry MA, Ousley PJ (2001). Deep lamellar endothelial keratoplasty in the first United States patients. *Cornea*.

[35] Price FW, Price MO (2005). Descemet’s stripping with endothelial keratoplasty in 50 eyes: a refractive neutral corneal transplant. *Journal of Refractive Surgery*.

[36] Gorovoy MS (2006). Descemet-stripping automated endothelial keratoplasty. *Cornea*.

[37] Melles GR, Ong TS, Ververs B, van der Wees J (2006). Descemet membrane endothelial keratoplasty (DMEK). *Cornea*.

[38] Arenas E, Esquenazi S, Anwar M, Terry M (2012). Lamellar corneal transplantation. *Survey of Ophthalmology*.

[39] Price MO, Price FW (2007). Descemet stripping with endothelial keratoplasty for treatment of iridocorneal endothelial syndrome. *Cornea*.

[40] Bromley JG, Randleman JB, Stone D, Stulting RD, Grossniklaus HE (2012). Clinicopathologic findings in iridocorneal endothelial syndrome and posterior polymorphous membranous dystrophy after Descemet stripping automated endothelial keratoplasty. *Cornea*.

[41] Pineda R, Jain V, Shome D, Hunter DC, Natarajan S (2010). Descemet’s stripping endothelial keratoplasty: is it an option for congenital hereditary endothelial dystrophy?. *International Ophthalmology*.

[42] Price MO, Price FW (2007). Descemet’s stripping endothelial keratoplasty. *Current Opinion in Ophthalmology*.

[43] Kobayashi A, Yokogawa H, Sugiyama K (2012). Clinical results of the Neusidl Corneal Inserter((R)), a new donor inserter for Descemet's stripping automated endothelial keratoplasty, for small Asian eyes. *Ophthalmic Surgery Lasers & Imaging*.

[44] Yokogawa H, Kobayashi A, Sugiyama K (2012). Clinical evaluation of a new donor graft inserter for Descemet’s stripping automated endothelial keratoplasty. *Ophthalmic Surgery Lasers & Imaging*.

[45] Price MO, Baig KM, Brubaker JW, Price FW (2008). Randomized, prospective comparison of precut vs surgeon-dissected grafts for Descemet stripping automated endothelial keratoplasty. *American Journal of Ophthalmology*.

[46] Terry MA, Shamie N, Chen ES, Phillips PM, Hoar KL, Friend DJ (2009). Precut tissue for Descemet’s stripping automated endothelial keratoplasty: vision, astigmatism, and endothelial survival. *Ophthalmology*.

[47] Sikder S, Nordgren RN, Neravetla SR, Moshirfar M (2011). Ultra-thin donor tissue preparation for endothelial keratoplasty with a double-pass microkeratome. *American Journal of Ophthalmology*.

[48] Phillips PM, Phillips LJ, Saad HA (2013). ‘Ultrathin’ DSAEK tissue prepared with a low-pulse energy, high-frequency femtosecond laser. *Cornea*.

[49] Baratz KH, McLaren JW, Maguire LJ, Patel SV (2012). Corneal haze determined by confocal microscopy 2 years after Descemet stripping with endothelial keratoplasty for Fuchs corneal dystrophy. *Archives of Ophthalmology*.

[50] van der Meulen IJE, van Riet TC, Lapid-Gortzak R, Nieuwendaal CP, van den Berg TJ (2012). Correlation of straylight and visual acuity in long-term follow-up of manual Descemet stripping endothelial keratoplasty. *Cornea*.

[51] Woodward MA, Raoof-Daneshvar D, Mian S, Shtein RM (2013). Relationship of visual acuity and lamellar thickness in Descemet stripping automated endothelial keratoplasty. *Cornea*.

[52] Yamaguchi T, Negishi K, Yamaguchi K (2010). Comparison of anterior and posterior corneal surface irregularity in Descemet stripping automated endothelial keratoplasty and penetrating keratoplasty. *Cornea*.

[53] Anshu A, Planchard B, Price MO, da RPC, Price FW (2012). A cause of reticular interface haze and its management after Descemet stripping endothelial keratoplasty. *Cornea*.

[54] Afshari NA, Gorovoy MS, Yoo SH (2012). Dislocation of the donor graft to the posterior segment in Descemet stripping automated endothelial keratoplasty. *American Journal of Ophthalmology*.

[55] McCauley MB, Price MO, Fairchild KM, Price DA, Price FW (2011). Prospective study of visual outcomes and endothelial survival with Descemet membrane automated endothelial keratoplasty. *Cornea*.

[56] Guerra FP, Anshu A, Price MO, Price FW (2011). Endothelial keratoplasty: fellow eyes comparison of Descemet stripping automated endothelial keratoplasty and Descemet membrane endothelial keratoplasty. *Cornea*.

[57] Parker J, Dirisamer M, Naveiras M (2012). Outcomes of Descemet membrane endothelial keratoplasty in phakic eyes. *Journal of Cataract and Refractive Surgery*.

[58] Rudolph M, Laaser K, Bachmann BO, Cursiefen C, Epstein D, Kruse FE (2012). Corneal higher-order aberrations after Descemet’s membrane endothelial keratoplasty. *Ophthalmology*.

[59] Dirisamer M, Parker J, Naveiras M (2013). Identifying causes for poor visual outcome after DSEK/DSAEK following secondary DMEK in the same eye. *Acta Ophthalmologica*.

[60] Dapena I, Ham L, Netuková M, van der Wees J, Melles GR (2011). Incidence of early allograft rejection after Descemet membrane endothelial keratoplasty. *Cornea*.

[61] Anshu A, Price MO, Price FW (2012). Risk of corneal transplant rejection significantly reduced with Descemet’s membrane endothelial keratoplasty. *Ophthalmology*.

[62] Naveiras M, Dirisamer M, Parker J (2012). Causes of glaucoma after Descemet membrane endothelial keratoplasty. *American Journal of Ophthalmology*.

